# Screening and Functional Analysis of tsRNAs Associated with Diabetic Foot Ulcer Tissues

**DOI:** 10.3390/biomedicines13122887

**Published:** 2025-11-26

**Authors:** Xiaona He, Yufei Chen, Lihong Liu, Siqi Fu, Yuyi Tian, Yihan Lin, Shang Zhu, Luhong Dai, Xiaojia Wen

**Affiliations:** 1 Department of Rehabilitation Medicine, The Second Xiangya Hospital of Central South University, Changsha 410073, China; 238212283@csu.edu.cn (X.H.); drliulh@csu.edu.cn (L.L.); 18801068875@163.com (Y.T.); linyihan3613@163.com (Y.L.); 238212282@csu.edu.cn (S.Z.); dailuhong22@163.com (L.D.); 2 Chengdu Fifth People’s Hospital, Chengdu 611130, China; chenyufei6677@163.com; 3 Department of Dermatology, The Second Xiangya Hospital of Central South University, Changsha 410073, China; fusiqi@csu.edu.cn

**Keywords:** diabetic foot ulcer, noncoding RNA, transcriptome sequencing, tsRNA, wound healing

## Abstract

**Background**: Diabetic foot ulcer (DFU) is a serious complication of diabetes mellitus, and its inability to heal is closely linked to vascular pathology. However, the specific molecular regulatory networks involved remain unclear. This study hypothesizes that tRNA-derived small RNAs (tsRNAs) may be associated with endothelial dysfunction in DFU. **Methods**: RNA sequencing (RNA-seq) was performed on DFU (*n* = 3) and healthy foot skin (*n* = 3) samples. Bioinformatics analysis identified differential expression of tsRNAs, with the ten most significantly differentially expressed tsRNAs validated by qRT-PCR. Target gene prediction and GO/KEGG enrichment analysis were then conducted on the four tsRNAs that demonstrated significant differential expression, as confirmed by qRT-PCR. **Results**: The results revealed that there were 49 differentially expressed tsRNAs between the two groups of samples. qRT-PCR validation confirmed that the expression trends of ten tRNAs were consistent with sequencing results. Among these, the 4770 potential target genes of four tRNAs exhibiting significant expression differences primarily encompassed the cell growth factor family and the Smad protein family. GO analysis revealed that the target genes were located mainly in the cytoplasm and organelle membranes and functioned by specifically binding to DNA in the transcriptional regulatory regions. KEGG pathway enrichment revealed that the differential tsRNAs were closely associated with pathways involved in cytoplasmic lysis and phagocytosis and the transforming growth factor beta (TGF-β) signaling pathway. **Conclusions**: This study systematically reveals the differential expression profile of tissue-specific tsRNAs in DFU tissue, thereby enriching the molecular pathological theory underlying the poor healing capacity of DFU. It also provides experimental evidence for the clinical translation of tsRNAs as early diagnostic markers for DFU.

## 1. Introduction

Diabetic foot ulcer (DFU) is a condition in which patients with diabetes mellitus develop neuropathy and/or peripheral arteriopathy of the lower extremities, which may manifest as infection, ulceration, or degradation of foot and ankle tissues. The prevalence of DFU is approximately 6.3% worldwide [1], and it is a leading cause of amputation [2]. Its high prevalence and disability rate impose a heavy burden on patients and society [3]. Therefore, new strategies to prevent and treat DFU are essential. The non-healing mechanism of DFU involves four core factors: peripheral vascular disease, neuropathy, infection, and immune dysfunction [4]. Among these, vascular endothelial dysfunction is considered the pivotal link initiating and sustaining chronic wounds [5,6]. Under physiological conditions, vascular endothelial cells regulate vasodilation and vasoconstriction by secreting endothelial nitric oxide synthase (eNOS) [7]. Vascular endothelial growth factor (VEGF) acts specifically on these cells to promote their proliferation and the formation of new blood vessels [8]. In diabetic patients, however, mechanisms such as the abnormal activation of the polyol pathway, oxidative stress, the excessive activation of protein kinase C (PKC) and the accumulation of protein glycosylation and advanced glycation end products induced by the hyperglycemic environment can lead to increased endothelial cell apoptosis and senescence, as well as reduced proliferation and migration capacity and impaired neovascularization [9].

Zhao et al. revealed through single-cell RNA-seq that endothelial cells exhibit significant heterogeneity within the DFU microenvironment: the EC-5 subpopulation is enriched in non-healing wounds and highly expresses the TNF-α (Tumor Necrosis Factor Alpha) signaling pathway, whereas the EC-8 subpopulation predominates in healing wounds and is closely associated with the activation of pro-repair pathways, including AMPK, PPAR, and PI3K/AKT/mTOR [10]. The findings of these studies suggest that imbalanced endothelial cell phenotypic switching and functional impairment are key mechanisms underpinning the chronicity of DFU. However, the comprehension of their upstream molecular regulatory networks, particularly post-transcriptional noncoding RNA (ncRNA) regulatory mechanisms, remains extremely limited.

In recent years, the role of epigenetic regulation in the pathogenesis of DFU has garnered significant attention. Prolonged hyperglycemia has been demonstrated to induce ‘metabolic memory’ through alterations in ncRNA expression, DNA methylation, and histone modifications. This, in turn, has been shown to persistently influence inflammatory responses, extracellular matrix (ECM) deposition, and angiogenesis [1]. Recent studies have demonstrated the regulatory role of microRNAs (miRNAs) and long noncoding RNAs (lncRNAs) in endothelial cell function and DFU healing [11,12]. However, the role of tsRNAs—a novel class of ncRNA—in the pathological process of DFU remains entirely unknown. The generation of tsRNA is a consequence of precise cleavage of tRNA, and this expression is subject to significant alterations when the cell is experiencing cellular stress [13]. It is evident that there are two distinct categories of tsRNAs: tRNA-derived fragments (tRFs) and stress-induced tRNA halves (tiRNAs). The distinguishing factor between these categories is the variable cleavage position at which they are produced [14]. tsRNAs have been demonstrated to participate in various disease processes, such as neurodegenerative diseases and cardiovascular diseases, by regulating gene transcription, translation, and epigenetic modifications [13]. However, studies on the relationship between DFU and tsRNAs at home and abroad are lacking. Given that endothelial dysfunction lies at the core of vascular pathology in diabetic foot ulcer, we hypothesize that tsRNA may be involved in the mechanisms associated with endothelial cell dysfunction.

Therefore, this study aims to utilize RNA-seq systems to map the differential expression profile of tRNA within DFU tissue, thereby investigating the potential regulatory role of tRNA in endothelial dysfunction associated with DFU.

## 2. Materials and Methods

### 2.1. Sampling of Participants and Organizations

In this study, three foot ulcer samples were obtained from DFU patients admitted to the Department of Orthopedics of the Second Xiangya Hospital of Central South University as the experimental group (group A) and coded A1, A2, and A3. The inclusion criterion for the experimental group was patients aged > 18 years who could be definitively diagnosed with DFU according to the 1999 World Health Organization diagnostic criteria. The exclusion criteria included patients with cancer or immune-related diseases, or patients who were receiving similar immunosuppressive drug therapy. Three control samples, B1, B2, and B3, were also collected from orthopedic patients at the Second Xiangya Hospital of Central South University during the same period; they formed the control group (group B). The inclusion criteria for the control group were as follows: age > 18 years, undiagnosed diabetes with no history or family history of diabetes, no tumors or autoimmune diseases, and no infections or bleeding disorders of the skin of the foot. The study was approved by the Ethics Committee of the Second Xiangya Hospital of Central South University (Ethics approval number: LYF2022156).

Sample size considerations: This study employed three biological replicates per group, primarily based on the following considerations: Firstly, obtaining clinical samples of deep skin tissue from diabetic foot ulcer is exceptionally challenging, requiring strict adherence to surgical indications and informed patient consent. Large-scale sample collection faces significant constraints in terms of ethics and feasibility. Secondly, preliminary pre-experiments demonstrated high intra-group consistency in tsRNA expression profiles, indicating that three biological replicates adequately reflect intra-group biological variation. Thirdly, RNA-seq technology itself exhibits high technical reproducibility, and this study employed stringent patient inclusion criteria (uniform Wagner staging, exclusion of infection interference), effectively reducing heterogeneity. Finally, the negative binomial model within the edgeR software (version 3.36.0) is specifically designed for small-sample RNA-seq datasets. Through empirical Bayesian estimation, it efficiently allocates information, enabling robust identification of differentially expressed molecules, even under *n* = 3 conditions [15]. Nevertheless, we acknowledge that the small sample size may limit sensitivity in detecting molecules with small effect sizes, a limitation addressed in the Discussion section.

### 2.2. RNA Extraction, Library Preparation, and RNA Sequencing

Total RNA was extracted from frozen tissues via the TRIzol method (Invitrogen, Carlsbad, CA, USA), followed by NanoDrop ND-1000 (Thermo Scientific, Wilmington, DE, USA) and agarose gel electrophoresis to determine the concentration, purity, and integrity of the extracted RNA. The extracted RNA was pretreated with the reagents included in the rtStar™ tRF&tiRNA pretreatment kit (Arraystar Inc., Rockville, MD, USA). A Multiplex Small RNA Library Prep Illumina Kit (Illumina, San Diego, CA, USA) was subsequently used for library construction, and the fragments in the mixed library were denatured with 0.1 M NaOH to generate single-stranded molecules, which were loaded into the reagent cartridge at a concentration of 1.8 pM. The Illumina NextSeq500 system (Illumina, San Diego, CA, USA) was subsequently used for standard small RNA-seq.

### 2.3. Data Quality Control and Screening

Raw sequence data were generated via Illumina NextSeq (Illumina, San Diego, CA, USA). Real-time base calling and mass filtering were used to generate clean reads, which were recorded in FASTq format, and the mass fraction of each sample was plotted via counts per million (CPM) to represent the gene expression level of the mapped reads. Differentially expressed tsRNAs were identified via the R package edgeR software (version 3.36.0), and a regression model with a negative binomial distribution was used to estimate *p*-values. The Benjamini–Hochberg method was employed for multiple testing correction to control the false discovery rate (FDR). Differences were considered statistically significant if the fold change (FC) was >1.5 and the *p*-value was <0.05.

### 2.4. Validation of Differentially Expressed Genes via Real-Time Fluorescence Quantitative PCR

After the RNA was pretreated, it was reverse transcribed into cDNA using the rtStar™ First-Strand cDNA Synthesis Kit (Arraystar Inc., Rockville, MD, USA). To detect each tsRNA, the cDNA template that was determined to express the gene in the previous step was selected for real-time PCR (Bio-Rad Laboratories, Hercules, CA, USA), thus validating the differential gene expression. We analyzed six samples and validated ten tsRNAs by qRT-PCR in total. Reference gene: U6 snRNA was validated for stability across all samples (CV = 0.58%). Replicates: Biological replicates *n* = 3 per group; each sample run in technical triplicate (Ct CV < 1.1%). Amplification efficiency: All primers were validated for amplification efficiency using a tenfold gradient dilution standard. The standard curve exhibited an R^2^ value > 0.998, with efficiencies ranging from 102.5% to 108.2%. (Supplementary Table S1).

### 2.5. Prediction of tsRNA Target Genes

The tsRNAs with significant expression differences verified by qRT-PCR were selected for the prediction of target genes via two software programs, Miranda v3.3a and TargetScan 8.0.

### 2.6. Bioinformatics and Functional Analysis of Differentially Expressed tsRNAs

To determine the potential biological functions of the selected tsRNAs, Gene Ontology (GO) accessed on 15 May 2022 and Kyoto Encyclopedia of Genes and Genomes (KEGG) Release 102.0 (1 April 2022) enrichment analyses were performed. Significantly enriched terms had *p*-values ≤ 0.05.

## 3. Results

### 3.1. RNA Concentration, Purity, and Integrity Testing

**Figure 1:**
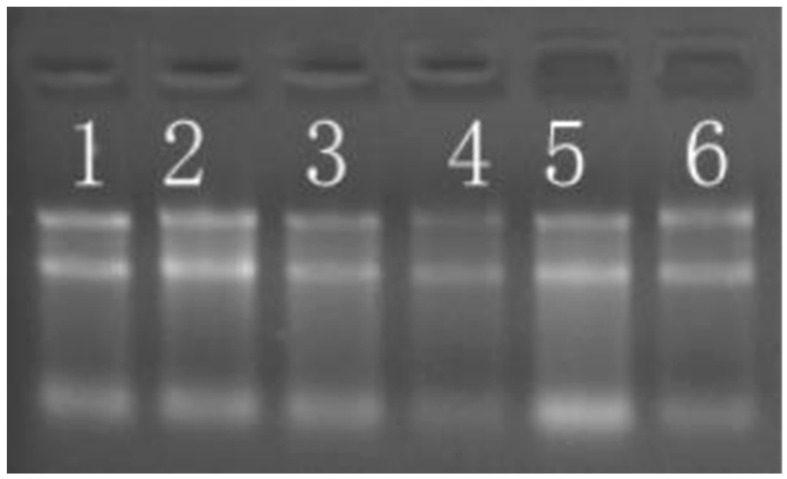
Agar gel electrophoresis was used to identify total RNA from both groups. Note: Bands 1–3 represent experimental samples A1, A2, and A3, and bands 4–6 represent control samples B1, B2, and B3.

All sample RNA qualities met high-throughput sequencing standards. Purity analysis revealed OD260/280 ratios > 2.0 and OD260/230 ratios > 1.8 across all samples, suggesting that the sample RNA was not contaminated with proteins, phenolics, or other impurities (Table 1). Furthermore, agar gel electrophoresis revealed clear and intact ribosomal RNA bands (Figure 1), which revealed that the extracted RNA was highly pure without excessive impurities or degradation and met the requirements of the experiment.

The sequencing libraries constructed in this study exhibited excellent quality, with all parameters meeting machine-processing standards. The information pertaining to the library preparation of each sample is delineated in Table 2. The lengths of libraries prepared from each sample were maintained between 134 base pairs (bp) and 160 bp (the length of the small RNAs after the removal of junctions ranged from 14 bp to 40 bp). The total library yield for all samples exceeded 10 ng, thereby satisfying the requirements for online sequencing.

### 3.2. Quality Control Results of the Sequencing Data

**Figure 2:**
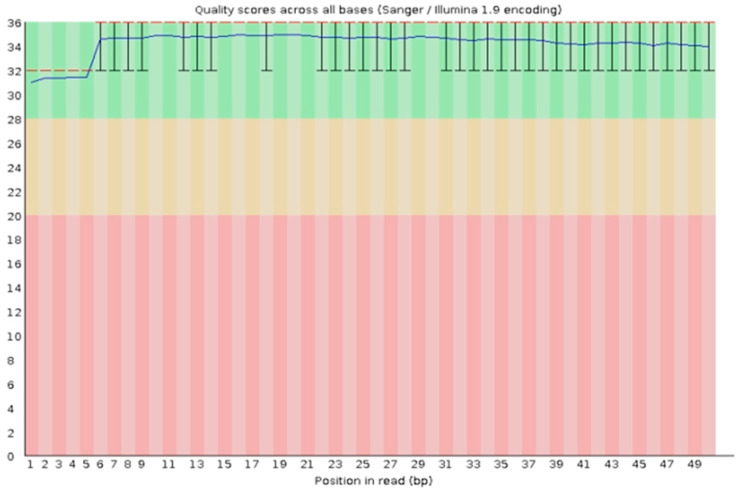
Base mass distribution diagram of the sample (using Sample A1 as an example). Note: Red region: Indicates extremely low base quality. Yellow region: Indicates moderate base quality. Green region: Indicates higher base quality, with highly reliable sequencing results.

A base quality distribution graph is presented in Figure 2. The horizontal axis indicates the base reading position, and the vertical axis indicates the size of the Q (Q = −10 log10(P)) value (quality fraction). The red line in the distribution graph indicates the median of the Q value, and the blue line indicates the average of the Q value. The quality benchmark was set at Q30 (corresponding to an error rate < 0.001 and accuracy > 99.9%). Table 3 shows the base quality of each sample. This indicates excellent sequencing quality, meeting requirements for subsequent analysis.

### 3.3. Expression of the tsRNAs in the Samples

**Figure 3:**
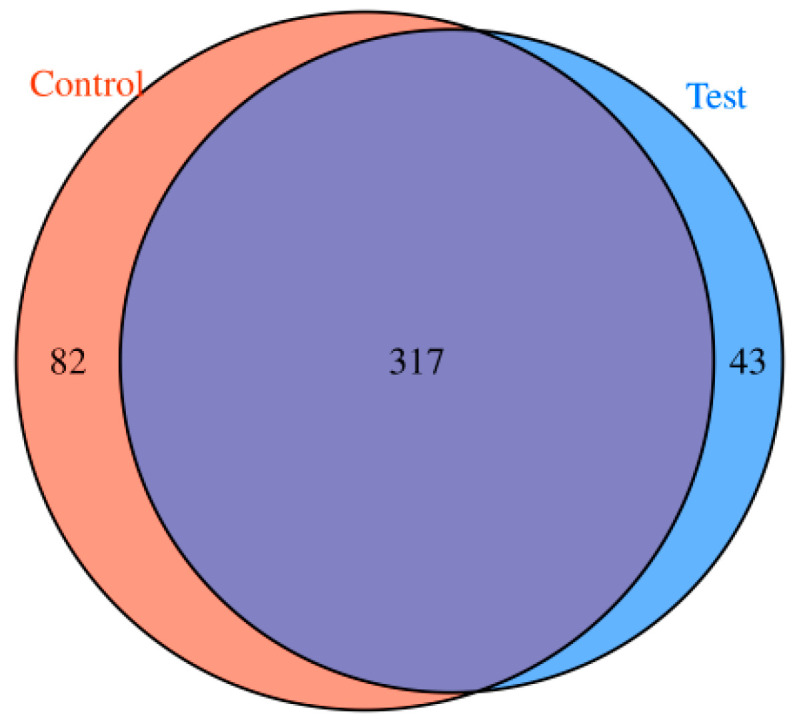
Venn diagram of tsRNAs expressed in the two groups of samples.

**Figure 4:**
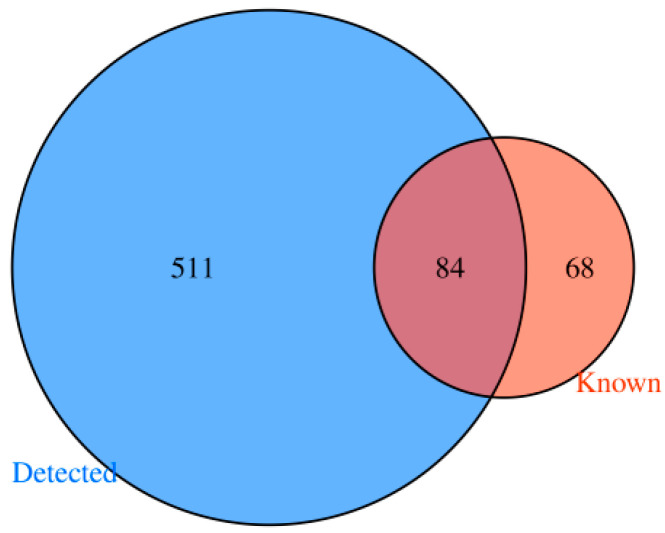
Venn diagram of tsRNAs expressed in the two groups of samples with tRFdb.

After the two groups of samples were sequenced, 360 and 399 tsRNAs were identified in the experimental group and the control group, respectively, and the two groups of samples had 317 common tsRNAs. Additionally, 43 tsRNAs were expressed in group A only, while 82 were uniquely expressed in group B, and the results were obtained and then presented in a Venn diagram (Figure 3). Moreover, the shared tsRNAs detected in the samples were compared to tRFs that are well known in the tRFdb database, and 84 of them were found to be present in the tRFdb database (Figure 4).

**Figure 5:**
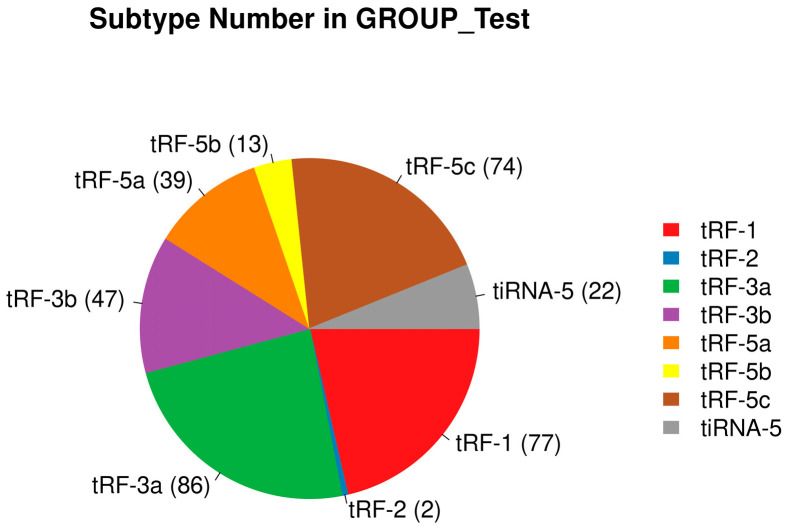
Distribution of tsRNA species in the experimental group.

**Figure 6:**
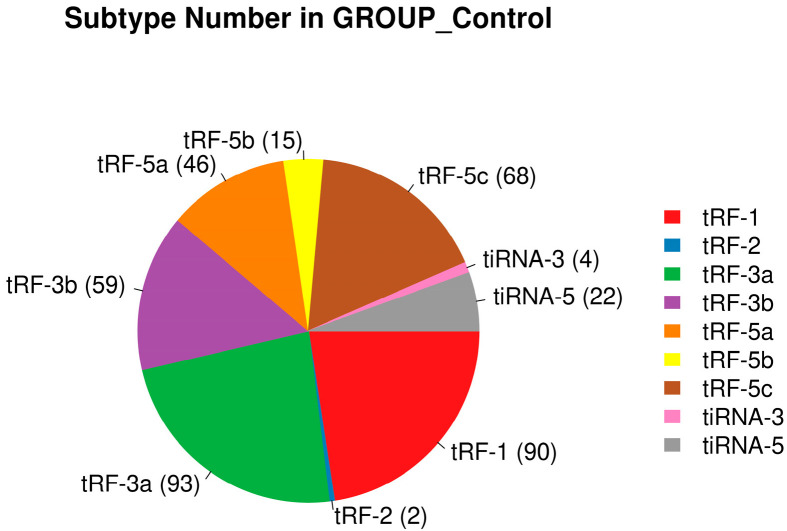
Distribution of tsRNA species in the control group.

In the DFU group, we detected a total of 77 tRF-1, 2 tRF-2, 88 tRF-3a, 47 tRF-3b, 39 tRF-5a, 13 tRF-5b, 74 tRF-5c, and 22 tiRNA-5. In the control group, we detected a total of 90 tRF-1, 2 tRF-2, 93 tRF-3a, 59 tRF-3b, 46 tRF-5a, 15 tRF-5b, 68 tRF-5c, 4 tiRNA-3, and 22 tiRNA-5. This result is presented in the form of pie charts (Figure 5 and Figure 6).

**Figure 7:**
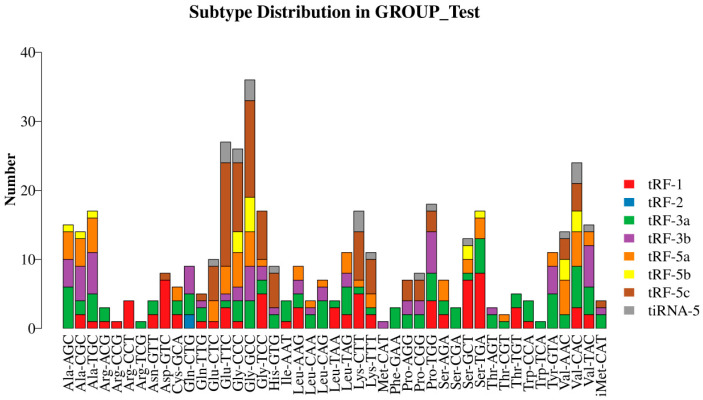
Stacking of various classes of tsRNAs in the experimental group in relation to the tRNAs from which they were derived.

**Figure 8:**
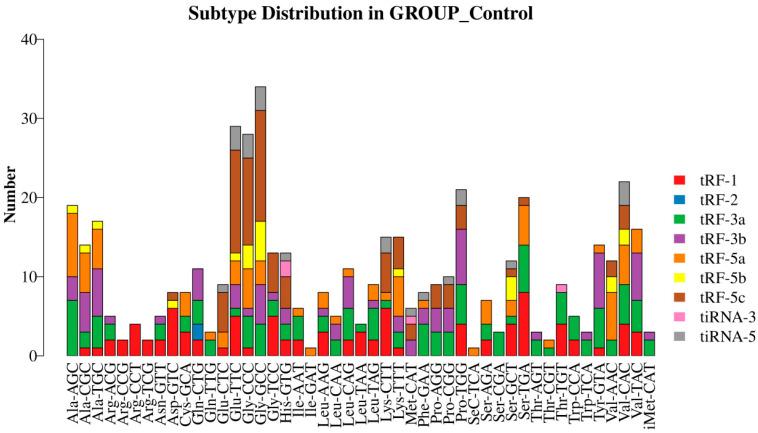
Stacking of various classes of tsRNAs in the control group in relation to the tRNAs from which they were derived.

A mature tRNA or its precursor tRNA can generate different tsRNA fragments via specific shearing. Therefore, in this study, we plotted the stacked bars of tsRNAs from different tRNA isoacceptors (i.e., tRNA species carrying the same amino acids but different anticodons) using the obtained tsRNA data (Figure 7 and Figure 8).

### 3.4. Differential Expression of tsRNAs in Samples from Both Groups

**Figure 9:**
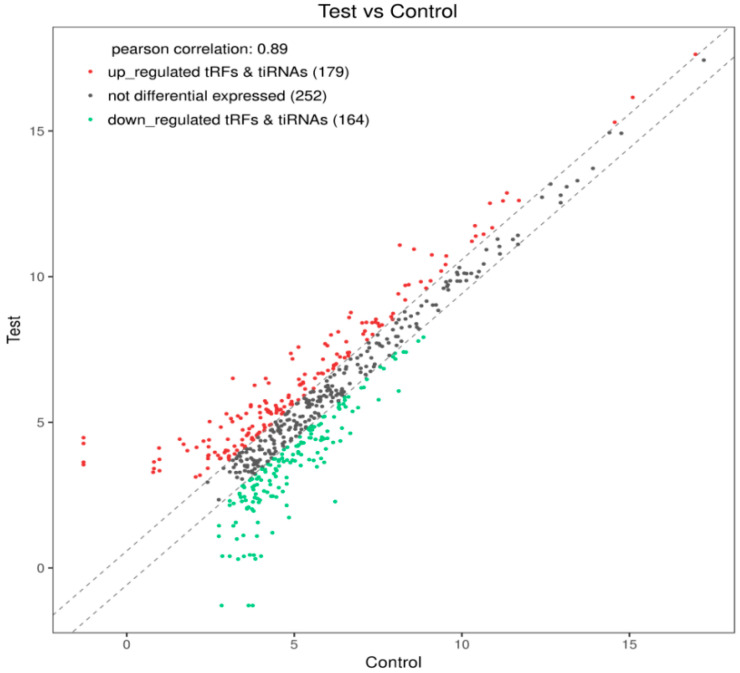
Scatter plot of differentially expressed tsRNAs in the two groups of samples, where the red and green dots represent the differentially expressed tsRNAs in the two groups (FC > 1.5). The gray dots represent the tsRNAs that are not significantly differentially expressed between the two groups.

To visualize the differential tsRNA expression in the two groups of samples, a scatter plot was drawn using the log_2_ of the tsRNA CPM values in the two groups as the axes (Figure 9), in which the red dots and the green dots represent the differentially expressed tsRNAs in the two groups (FC > 1.5). The gray dots represent the tsRNAs that are not significantly differentially expressed between the two groups. A total of 179 upregulated and 164 downregulated tsRNA fragments were identified in the experimental group relative to the control group.

**Figure 10:**
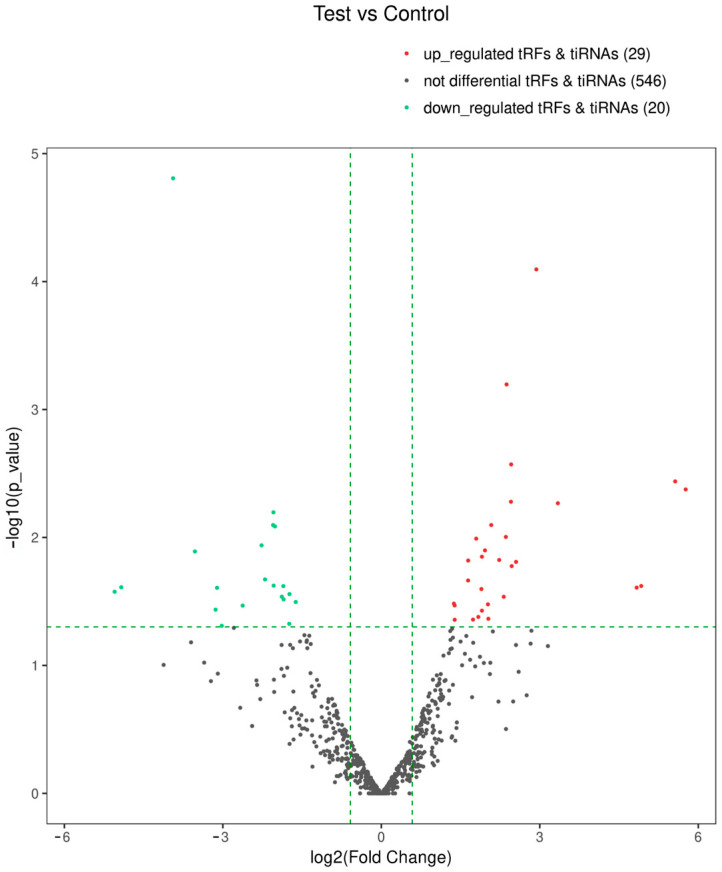
Volcano plot of the differentially expressed tsRNAs in the two groups of samples, where the red and green dots represent the differentially expressed tsRNAs between the two groups according to the FC > 1.5 and *p* < 0.05 criteria. The gray dots represent the tsRNAs that are not significantly differentially expressed between the two groups.

Moreover, volcano plots were drawn, with $\log_{2}FC$ as the horizontal coordinate and −$\log_{10}$ *p*-value as the vertical coordinate of each fragment between the two groups (Figure 10), which visualized the changes in the expression of the tsRNAs in the two groups more intuitively and visually. Forty-nine differentially expressed tsRNAs with FC > 1.5 and *p* < 0.05 were identified as significantly different, 29 of which were upregulated, and 20 were downregulated in the experimental group relative to the control group.

The significantly differentially expressed tsRNAs are shown in Table 4 and Table 5.

**Figure 11:**
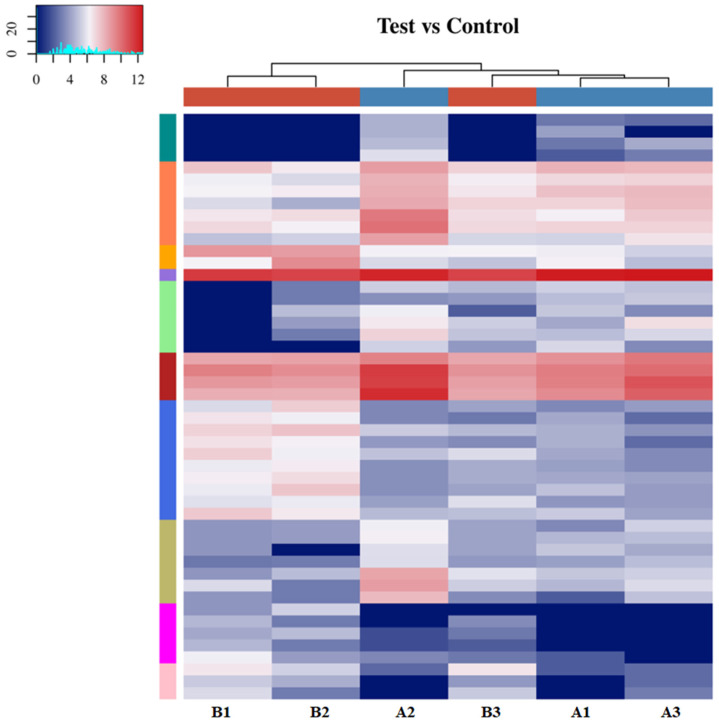
Heatmap of clustering of tsRNAs between samples. (1) For color classes, red represents above-average expression values, and blue represents below-average expression values. (2) The top bar colors represent sample groupings. (3) The left colored bars indicate divisions via k-means.

In this study, samples were grouped via cluster analysis, and a hierarchical cluster plot was drawn (Figure 11). In the clustering diagram, the six vertical columns represent six samples, each row represents a tsRNA, and the color in the diagram represents the relative expression level of that tsRNA. The clustering heatmap revealed different tsRNA expression profiles among several samples, with significant differences between the expression profiles of the experimental and control groups.

### 3.5. Validation of Sequencing Results via qRT-PCR

**Figure 12:**
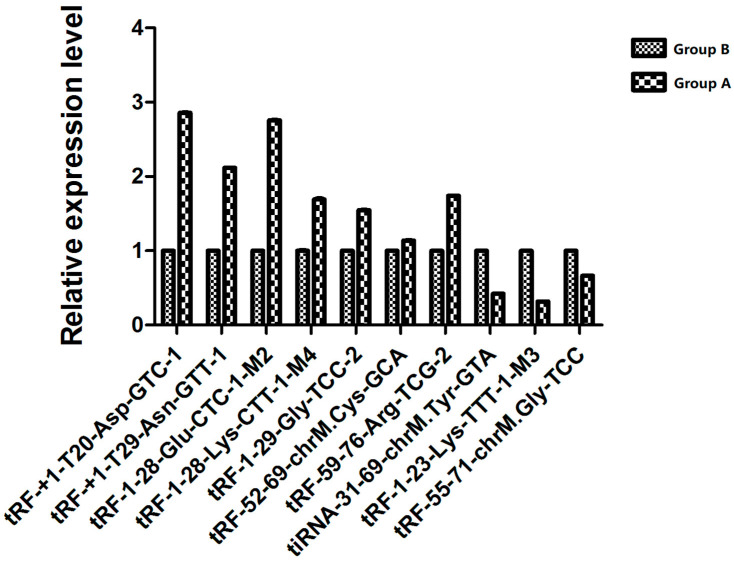
Relative expression results of selected tsRNAs in the two groups via qRT-PCR.

Seven tsRNAs whose expression was significantly upregulated in group A relative to that in group B were selected (tRF-+1:T20-Asp-GTC-1, tRF-+1:T29-Asn-GTT-1, tRF-52:69-chrM.Cys-GCA, tRF-1:28-Lys-CTT-1-M4, tRF-59:76-Arg-TCG-2, tRF-1:28-Glu-CTC-1-M2 and tRF-1:29-Gly-TCC-2), whereas tsRNAs with relatively significant downregulation were selected (tRF-1:23-Lys-TTT-1-M3, tiRNA-31:69-chrM. Tyr-GTA and tRF-55:71- chrM. Gly-TCC) for validation via qRT-PCR. The results were similar to the sequencing results. The relative expression levels of the screened tsRNAs between the two groups are displayed in Figure 12, which shows that tRF-+1-T20-Asp-GTC-1, tRF-+1-T29-Asn-GTT-1, and tRF-1-28-Glu-CTC-1-M2 were significantly upregulated in group A relative to group B and were consistent with the sequencing results, whereas tiRNA-31-69-chrM. Tyr-GTA was downregulated significantly relative to that in group B, and this trend was consistent with the sequencing results. However, although tRF-1-28-Lys-CTT-1-M4, tRF-1-29-Gly-TCC-2, tRF-52-69-chrM. Cys-GCA, tRF-59-76-Arg-TCG-2, tRF-55-71-chrM, and Gly-TCC tended to be upregulated or downregulated, which is consistent with the sequencing results, qRT-PCR validation data revealed that the differences in expression were not statistically significant (*p*-values of 0.52, 0.34, 0.86, 0.44, and 0.50, respectively).

### 3.6. Prediction of Target Genes

**Figure 13:**
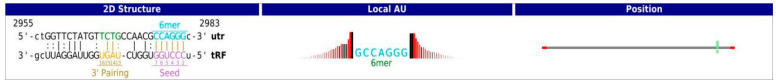
Schematic representation of the tsRNA seed sites and the target mRNA binding sites (with TGF-β2 as an example).

**Figure 14:**
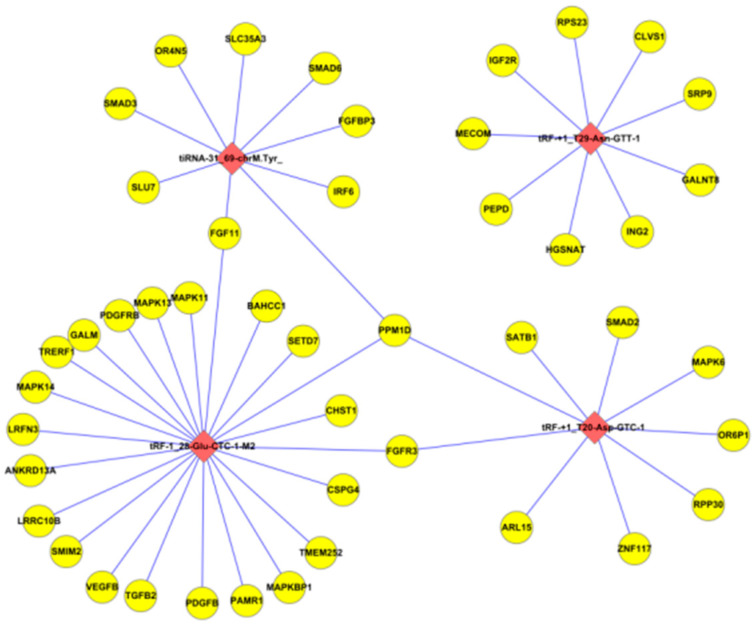
Network diagram of the validated tsRNAs with several potential target genes. Note: pink diamonds represent tsRNAs, and yellow circles represent potential target genes.

tsRNAs contain a seed sequence within them that may silence mRNAs via complementary base pairing with target mRNAs. In this study, TargetScan (v8.0) and miRanda (v3.3a) software were used to analyze the predicted target genes of the four tsRNAs that were significantly differentially expressed according to the qRT-PCR validation (tRF-+1-T20-Asp-GTC-1, tRF-+1-T29-Asn-GTT-1, tRF-1-28-Glu-CTC-1-M-2, and tiRNA-31-69-chrM. Tyr-GTA) (Figure 13). The total number of potential target genes predicted for the four tsRNAs was 4770, of which 838 target genes were predicted for tRF-+1-T20-Asp-GTC-1, 476 target genes were predicted for tRF-+1-T29-Asn-GTT-1, 2842 target genes were predicted for tRF-1-28-Glu-CTC-1-M2, and 1188 target genes were predicted for tiRNA-31-69-chrM. Tyr-GTA. There were 464 target genes that were predicted for two or more different tsRNAs. After effective screening, the association network of some target genes with the four tsRNAs was mapped (Figure 14). The target genes screened by TargetScan and miRanda encoded a variety of cell growth factors, including platelet-derived growth factor (PDGF), VEGF, fibroblast growth factor (FGF), transforming growth factor beta (TGF-β), and insulin-like growth factor (IGF) (Table 6).

### 3.7. GO Analysis of Target Genes

**Figure 15:**
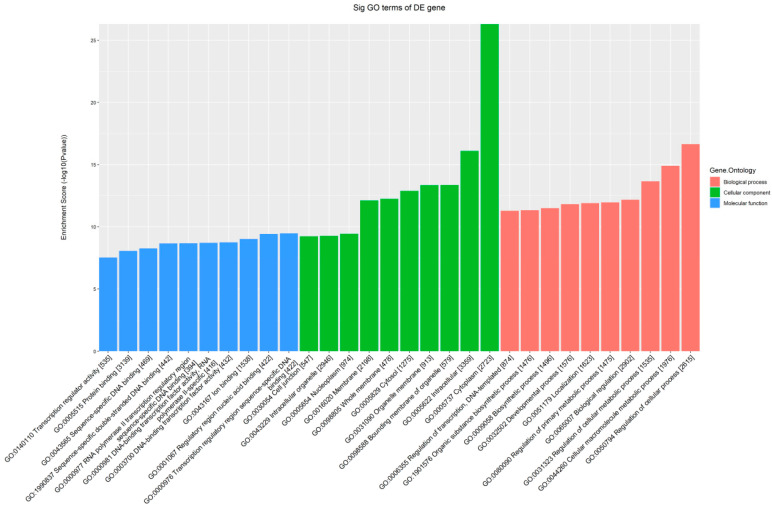
Bar graph of the GO enrichment results of the target genes. Note: Red represents the biological process category, green represents the cellular composition category, and blue represents the molecular function category. To make the values easy to observe, the enrichment degree is denoted by −$\log_{10}$ *p*-value.

The GO analysis of all the predicted target genes revealed that 2615 terms were enriched in the target genes, with “regulation of cellular processes” (GO: 0050794) being the most significantly enriched biological process term and “cytoplasm” (GO: 0005737) being the most significantly enriched cellular component term. The most significantly enriched molecular function term was “Transcription regulatory region sequence-specific DNA binding” (GO: 0030054), which was enriched in a total of 442 target genes. A bar graph of the enrichment level of target genes in the GO database was created using the GO entry name as the horizontal axis and the enrichment value as the vertical axis (Figure 15).

.

### 3.8. KEGG Pathway Analysis of Target Genes

**Figure 16:**
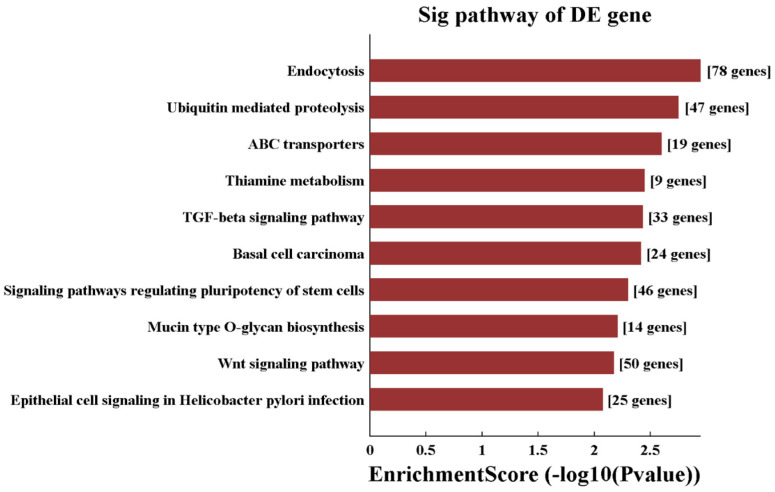
Histogram of KEGG pathways associated with the differentially expressed tsRNAs. Note: *p*-values are calculated as −$\log_{10}$ *p*-value for visualization of values.

We performed and KEGG pathway analysis of the target genes and identified 32 enriched pathways associated with the differentially expressed tsRNAs [16,17]. We plotted the histogram of the top ten pathways with enrichment scores versus −$\log_{10}$ *p*-value (Figure 16), among which the pathway with the highest enrichment score was the pathway related to “cellular phagocytosis and cytophagy” (pathway ID: 04144), and 78 target genes were involved in this pathway. The second most enriched pathway was the ubiquitin-mediated protein hydrolysis pathway (pathway ID: hsa04120), with 47 target genes involved in this pathway.

## 4. Discussion

Chronic diabetic foot ulcer is often difficult to heal [18], greatly affecting the physical and mental health of patients. Previous studies have shown that the pathogenesis of DFU disease is related mainly to peripheral vascularization and neuropathy [19], but the exact mechanism is still unknown. Researchers have now begun to explore the potential link between DFU and small-molecule noncoding RNAs [12,20]. However, studies comparing tsRNAs and DFU have not yet been reported.

The present study was the first to identify 363 differentially expressed tRNAs via high-throughput sequencing. Among the 49 tRNAs that exhibited significant differential expression (*p* < 0.05), four key molecules were validated: three were found to be upregulated (tRF-+1-T20-Asp-GTC-1, tRF-+1-T29-Asn-GTT-1, tRF-1-28-Glu-CTC-1-M2); one was downregulated (tiRNA-31-69-chrM.Tyr-GTA). These tsRNAs, by targeting 4770 candidate genes, demonstrated significant enrichment in functional categories including ‘regulation of cellular metabolic processes,’ ‘transcription factor activity,’ and ‘RNA polymerase II-specific domains.’ The analysis revealed their profound involvement in 32 signaling pathways, including phagocytosis, the ubiquitin–proteasome pathway, TGF-β, Wnt, and stem cell pluripotency regulation. This work organically integrates ncRNA research with the structural foundations of vascular pathophysiology, providing a novel molecular framework for understanding the mechanisms underlying DFU non-healing.

The subsequent sections of this study discuss the core findings, clinical implications, and study limitations.

### 4.1. Core Findings of the Study

The target genes screened by TargetScan and miRanda encode the PDGF, VEGF, FGF, TGF-β, and IGF proteins, which are cell growth factors. These cell growth factors can facilitate wound repair through their involvement in the proliferation and migration of endothelial cells, keratinized cells, and fibroblasts [21,22] and promote vascular endothelial regeneration and chemotaxis [23,24]. The wound healing process of DFU involves the proliferation of granulation tissue, vascularization of skin tissue, and epidermal hyperplasia, but this process is often accompanied by infections and deficiencies in cellular growth factors, such as PDGF, VEGF, and FGF, which lead to delayed healing of DFU wounds [25,26]. VEGF belongs to a family of structurally related proteins, with a gene length of 14 kilobases located on chromosome 6p21.3, which contains eight exons and seven introns in its structure [27]. This family of cell growth factors promotes vascular regeneration during wound healing [28]. FGF plays important roles in neural development, angiogenesis, and wound healing [29,30]. Dysregulation of FGF signaling has been shown to be closely associated with cardiovascular disease development [31]. Some miRNAs and lncRNAs are involved in regulating FGF signaling and various cellular processes. Dysregulation of FGF signaling not only directly causes human diseases but also indirectly contributes to the development of diseases by affecting angiogenesis and immune function [32]. The proliferation and differentiation of endothelial progenitor cells constitute a major part of angiogenesis in diabetic feet with chronic ulcers [33], and FGF2 is involved in the regulation of angiogenesis through fibroblast growth factor receptor 1/2 (FGFR1/2) signaling. FGF2 can promote the proliferation and migration of endothelial cells through FGFR1/2 signaling [34] and the secretion of VEGF/ANGPT2 (Angiopoietin2, ANGPT2). FGF is often released from damaged wounds, and FGF1 and FGF2 may be involved in the inflammatory response through the chemotaxis of neutrophils to damaged tissues via FGFR2 [35]. However, despite the widespread recognition of these growth factors’ functions, the post-transcriptional regulatory mechanisms underlying their dysregulated expression in DFU remain unclear.

Recent research has revealed that tsRNAs can exert a ‘translation brake’ effect by interfering with translation initiation factors or ribosomal function [36]. Specifically, certain Ψ-bearing 5’ tsRNA can inhibit mRNA translation initiation by interacting with the eIF4F complex and the polyadenylate-binding protein cytoplasmic 1 (PABPC1) [37]. Under stressful conditions, tsRNAs can also bind directly to the ribosomal small subunit, thereby impeding peptide bond formation and globally inhibiting protein synthesis [38].

Clinical studies indicate that diabetic foot ulcers exhibit defective growth factor and receptor expression, including significant VEGF [39], FGF [40], and FGFR [41] downregulation. This insufficient growth factor signaling is closely associated with impaired proliferation of vascular endothelial cells in the wound, impaired angiogenesis, and delayed healing [42]. Based on the aforementioned evidence, we propose the following hypothesis for verification: The tsRNAs upregulated in the DFU group (e.g., tRF-+1-T20-Asp-GTC-1, tRF-+1-T29-Asn-GTT-1, and tRF-1-28-Glu-CTC-1-M2) may further inhibit the translation of VEGF/FGF and their receptor proteins during the progression of tissue injury. This, in turn, exacerbates impaired endothelial proliferation activity and vascular maturation dysfunction. This mechanism may represent a secondary blow occurring against a background of chronic hypoxia.KEGG pathway analysis of the potential target genes revealed a high enrichment score for the TFG-β signaling pathway and the presence of smad2 and smad3, an important family of proteins in the TGF-β signaling pathway, in the list of target genes of the differentially expressed tsRNAs. Smad proteins are a class of genes that are closely associated with wound healing and fibrosis [43]. Therefore, the results of the present study suggest that the differentially expressed tsRNAs may be involved in the regulation of DFU wound healing through the TGF-β/Smad signaling pathway. As demonstrated by previous studies, under conditions of stress (e.g., hypoxia or oxidative stress), tRNA can be specifically cleaved by nucleases such as angiopoietin (ANG) or Dicer, thereby generating tsRNA [44]. This ‘stress-responsive’ characteristic suggests that it may exert dynamic regulatory functions within the hyperglycemic microenvironment of diabetes. The present study reveals that tRF-+1-T20-Asp-GTC-1 is significantly upregulated in DFU groups, with its predicted target genes markedly enriched in the transforming growth factor-beta/Smad signaling pathway. This pathway exhibits bidirectional regulatory properties in wound healing: on the one hand, TGF-β/Smad signaling promotes fibroblast activation and extracellular matrix deposition [45]; WDR74-mediated activation of the TGF-β/Smad pathway has been demonstrated to improve diabetic ulcer healing [46]. Conversely, persistent overactivation of the TGF-β/Smad pathway leads to the formation of hypertrophic scars [47]. The Smad3 knockout mouse model has been used to demonstrate that the attenuation of TGF-β/Smad3 signaling accelerates keratinocyte differentiation and shortens healing time [48]. The present study hypothesizes that differentially expressed tsRNAs may dynamically intervene in the homeostatic balance between pro-repair and anti-repair processes by precisely regulating the translational efficiency of Smad proteins.

Endothelial cells fulfil two functions: firstly, they form the lining of blood vessels [49], and secondly, they act as central regulators within the metabolic-immune-coagulation network [50]. A high-glucose environment has been demonstrated to cause damage to the endothelial glycocalyx and degradation of tight junction proteins, such as VE-cadherin, leading to abnormal vascular permeability and inflammatory infiltration [51]. The differentially expressed tsRNA target genes identified in this study were significantly enriched in the ‘organelle membranes’ and ‘cytoplasm’ compartments, suggesting that tsRNAs may influence vascular structural integrity by regulating subcellular organelle functions (such as mitochondrial metabolic reprogramming and endoplasmic reticulum stress). It is noteworthy that the downregulated tiRNA-31-69-chrM.Tyr-GTA (a mitochondrial-derived tiRNA) may participate in the ‘mitochondrial dysfunction–endothelial apoptosis’ signaling axis, consistent with literature reports of mitochondrial tsRNAs regulating cell survival under hypoxic stress [52].

Neuropathy in DFU has been shown to result in sensory loss and to exacerbate ischemia by disrupting neurogenic vasodilatory signals (e.g., substance P, CGRP) [53]. The differentially expressed tsRNA target genes identified in this study are enriched in the Wnt signaling pathway, which plays a pivotal role in neurovascular interactions [54]. It is hypothesized that differential tsRNAs may influence neurovascular coupling factors such as Wnt7a/b, thereby affecting both neuroregeneration and angiogenesis. This phenomenon, known as a ‘neurovascular double-hit’ effect, provides a more comprehensive explanation than previous single-factor models.

Furthermore, the pathways with the highest enrichment scores in this study were phagocytosis and autophagy. Efficient clearance of necrotic tissue and apoptotic cells is crucial for chronic wound healing, a process that depends on the phagocytic function of macrophages [55]. Therefore, the differentially expressed tsRNAs that were found to be upregulated in the DF group may contribute to the persistent deterioration of the wound microenvironment by inhibiting the ‘clearance system’ synergistically, which warrants future validation.

### 4.2. Clinical Significance: Potential Diagnostic Markers

In light of the aforementioned mechanisms, this study demonstrates clear potential for clinical translation, primarily in the domain of diagnostic biomarker development. The three tsRNAs that have been verified by qRT-PCR as being upregulated in the DFU group are deemed suitable as risk stratification markers for DFU. Specifically, elevated expression of tRF-+1-T20-Asp-GTC-1, tRF-+1-T29-Asn-GTT-1, and tRF-1-28-Glu-CTC-1-M2 may predict non-healing risk, enabling early identification of high-risk patients and guiding personalized monitoring.

### 4.3. Research Limitations and Future Directions

The present study is subject to the following limitations:

1. The mechanism validation process is inadequate in terms of its depth. Although the differential expression of tsRNA has been verified via qRT-PCR, further confirmation is required to substantiate the direct interaction between tsRNA and target genes. This can be achieved through RNA pull-down, Argonaute Crosslinking and Immunoprecipitation Sequencing, and functional rescue experiments using tsRNA mimics/inhibitors. Future research may utilize endothelial-specific tsRNA transgenic animal models (e.g., conditional knockout/knock-in driven by VE-cadherin-Cre) to validate these hypotheses.
2. Limited sample size: The study’s reliance on a limited sample size can be attributed to the inherent challenges in acquiring clinical DFU patient skin ulcer tissue. It is evident that future research in this field should be accompanied by an expansion of sample sizes, in addition to the implementation of longitudinal cohort studies.
3. Lack of causal temporal verification: This study merely establishes a correlation between tsRNA and DFU, without confirming causality. Subsequent research should employ time-series analysis to clarify the temporal sequence between changes in tsRNA expression and endothelial dysfunction/cell death. Source tracing should also be conducted to determine whether tsRNA primarily originates from the release of necrotic cells.

In summary, this study investigated the relationship between tsRNA and diabetic foot ulcers (DFU). Findings indicate that three tsRNA molecules (tRF-+1-T20-Asp-GTC-1, tRF-+1-T29-Asn-GTT-1, and tRF-1-28-Glu-CTC-1-M2) were upregulated in the diabetic group, showing promise as biomarkers for predicting ulcer non-healing risk and monitoring treatment efficacy. However, comprehensive mechanistic validation and expanded clinical cohorts are essential next steps towards clinical translation.

## 5. Conclusions

This study identified differentially expressed tsRNA profiles in tissue from DFU, with their target mRNAs being significantly enriched in key angiogenesis pathways (VEGF, FGF, etc.). We propose that these tsRNAs may serve as potential biomarkers of tissue injury, reflecting the pathological state of endothelial dysfunction in DFUs through their association with post-transcriptional regulation of angiogenesis-related genes. However, existing data are insufficient to establish a causal role. This discovery provides new insights into the molecular mechanisms underlying DFU non-healing. Nevertheless, further clarification is needed on the precise timing of elevated tsRNA expression within the wound microenvironment, its origin (i.e., whether it is due to live cell stress responses or release from dead cells), and its temporal relationship with chronic hypoxia. Future research will focus on four key areas: (1) multi-omics integration: combining RNA-seq, proteomics, and single-cell sequencing to clarify the temporal relationship between tsRNA expression, tissue injury severity, and endothelial cell apoptosis/stress states, and to identify endothelial cell subpopulation-specific tsRNAs; (2) functional mechanism validation: using endothelial cell and diabetic animal wound models, conduct in vitro gain-of-function and loss-of-function experiments to determine whether the regulation of angiogenesis by key tsRNAs is driven by injury or is an independent phenomenon, while tracking dynamic changes within hypoxic and inflammatory microenvironments; (3) microenvironmental regulatory mechanisms: investigating the regulation of vascular endothelial-stromal interactions and intercellular communication by tsRNAs under hypoxic and inflammatory microenvironmental conditions, with a specific focus on analyzing their origin (active secretion vs. passive release) and colocalization characteristics with cell death markers; (4) clinical translational research: expanding the sample size to a prospective cohort of more than 100 patients to validate the correlation between tsRNA expression and clinical outcomes (e.g., healing time and amputation rate) and assess its diagnostic efficacy as an early warning biomarker.

In conclusion, this study establishes the foundation for understanding the tsRNA regulatory mechanisms in DFU. However, achieving clinical translation requires systematic functional validation and multidimensional integrated analysis to advance the development of precision diagnostic and therapeutic strategies targeting tsRNA.

## Figures and Tables

**Table 1:** Results of RNA quality control.

Sample ID	OD260/280	OD260/230	Conc. (ng/μL)	Volume (μL)	Quantity (ng)	QC Purity
A1	2.03	1.98	1453.29	60	87,197.40	Pass
A2	2.01	2.07	1415.11	60	84,906.60	Pass
A3	2.06	1.97	1017.77	120	122,132.40	Pass
B1	2.02	1.98	833.96	20	16,679.20	Pass
B2	2.03	2.05	1552.26	40	62,090.40	Pass
B3	2.03	2.09	1044.41	80	83,552.80	Pass

**Table 2:** Statistical results of the library preparation for each sample using an Agilent Bioanalyzer 2100.

Sample Name	Size (bp)	Conc. (ng/μL)	Conc. (nmol/L)	Volume (μL)	Total Amount (ng)
A1	147	3.77	38.8	10	37.7
A2	148	4.52	46.4	10	45.2
A3	149	1.61	16.4	10	16.1
B1	151	1.68	16.8	10	16.8
B2	151	1.22	12.3	10	12.2
B3	150	2.88	29.2	10	28.8

Note: Sample Name: sample name, Size: library length (after connectors are attached), Conc: library concentration, Volume: library volume, Total Amount: total amount of library.

**Table 3:** Quality of bases in each sample.

Sample	TotalRead	TotalBase	BaseQ30	BaseQ30 (%)
A1	401,434,350	8,028,687	378,499,462	94.29
A2	431,849,050	8,636,981	407,506,919	94.36
A3	377,755,450	7,555,109	356,416,302	94.35
B1	309,280,350	6,185,607	290,186,379	93.83
B2	464,885,000	9,297,700	437,691,659	94.15
B3	408,945,150	8,178,903	386,208,807	94.44

Note: TotalRead: total raw sequencing reads, TotalBase: total bases; BaseQ30: number of bases with Q > 30; BaseQ30%: proportion of bases with Q ≥ 30.

**Table 4:** Catalog of the top ten tsRNAs whose expression was upregulated in the experimental group compared with the control group.

tRF_ID	Type	Length	Fold_Change	*p*_Value	q_Value
tRF-+1:T20-Asp-GTC-1	tRF-1	20	54.26089443	0.004212478	0.40154022
tRF-+1:T29-Asn-GTT-1	tRF-1	29	47.28000655	0.003645393	0.40154022
tRF-54:74-Gly-GCC-1	tRF-3b	21	30.28283432	0.023949888	0.485617514
tRF-52:69-chrM.Cys-GCA	tRF-3a	18	28.51790893	0.024652481	0.485617514
tRF-1:28-Lys-TTT-3-M2	tRF-5c	28	10.1414708	0.00539886	0.40154022
tRF-59:76-Arg-TCG-2	tRF-3a	18	5.857601639	0.015515376	0.439602312
tRF-1:29-Glu-TTC-1	tRF-5c	29	5.532398325	0.016745139	0.452879889
tRF-1:28-Lys-CTT-1-M4	tRF-5c	28	5.493162564	0.002685946	0.399534518
tRF-1:29-Gly-TCC-2	tRF-5c	29	5.480706325	0.005259457	0.40154022
tRF-1:28-Glu-CTC-1-M2	tRF-5c	28	5.17343517	0.000636592	0.126257502

**Table 5:** Catalog of the top ten tsRNAs whose expression was downregulated in the experimental group compared with the control group.

tRF_ID	Type	Length	Fold_Change	*p*_Value	q_Value
tRF-1:23-Lys-TTT-1-M3	tRF-5b	23	33.10558868	0.026554554	0.493748731
tiRNA-31:69-chrM.Tyr-GTA	tiRNA-3	39	30.34609191	0.024494333	0.485617514
tiRNA-32:71-chrM.His-GTG-M1-39:C>A	tiRNA-3	40	15.36733852	1.55864 × 10^−5^	0.009273936
tRF-55:71-chrM.Gly-TCC	tRF-3a	17	11.54586865	0.012861814	0.439602312
tRF-+1:T23-chrM.Glu-TTC	tRF-1	23	8.801582905	0.036665962	0.516539645
tiRNA-32:70-chrM.His-GTG-M1-38:C>A	tiRNA-3	39	8.635336018	0.024745156	0.485617514
tRF-60:76-chrM.Asn-GTT	tRF-3a	17	8.111675029	0.048995367	0.588791763
tRF-1:16-Ala-AGC-9	tRF-5a	16	6.167279228	0.034074065	0.494489482
tiRNA-1:31-chrM.Met-CAT	tiRNA-5	31	4.814069244	0.011526844	0.439602312
tRF-58:74-chrM.Phe-GAA	tRF-3a	17	4.597817002	0.021279055	0.485617514

**Table 6:** Expression of several important cell growth factors and their receptors in target genes.

TsRNA	GeneSymbol	Context+	Structure	Energy
tRF-1_28-Glu-CTC-1-M2	VEGFB	−0.613	597	−123.669997
tRF-1_28-Glu-CTC-1-M2	FGF11	−0.293	140	−30.190001
tRF-1_28-Glu-CTC-1-M2	FGF19	−0.23	153	−30.59
tRF-1_28-Glu-CTC-1-M2	FGFR1	−0.188	154	−25.82
tiRNA-31_69-chrM.Tyr_	FGF7	−0.188	148	−33.779999
tRF-1_28-Glu-CTC-1-M2	FGFR3	−0.177	157	−28.450001
tRF-+1_T20-Asp-GTC-1	FGFR3	−0.176	145	−24.99
tiRNA-31_69-chrM.Tyr_	FGF11	−0.146	156	−17.879999
tRF-1_28-Glu-CTC-1-M2	PDGFB	−0.128	157	−20.82
tRF-1_28-Glu-CTC-1-M2	PDGFRB	−0.122	144	−26.690001
tRF-+1_T29-Asn-GTT-1	IGF2R	−0.109	160	−20.35
tRF-1_28-Glu-CTC-1-M2	IGF2BP2	−0.109	144	−21.549999
tRF-1_28-Glu-CTC-1-M2	TGFB2	−0.104	159	−28.6

Note: context+ stands for the Targetscan (v8.0) software threshold setting; the smaller it is, the better. Structure sets the score for miRanda; the larger the score is, the better. The lower the energy used to set the free energy for miRanda, the better.

## Data Availability

The datasets used and analyzed in this study are available from the corresponding author upon reasonable request.

## Supplementary Materials

The following supporting information can be downloaded at: https://www.mdpi.com/article/10.3390/biomedicines13122887/s1, Table S1: Calculation of qPCR Amplification Efficiency.

## Abbreviations

The following abbreviations are used in this manuscript:

DFU	Diabetic foot ulcer
RNA-seq	RNA sequencing
eNOS	Endothelial Nitric Oxide Synthase
bp	Base pair
CPM	Counts per million
FDR	False discovery rate
FC	Fold change
PKC	Protein kinase C
tsRNA	tRNA-derived small RNA
tRF	tRNA-derived fragment
tiRNAs	tRNA halves
tRNA	Transfer RNA
miRNA	microRNA
ncRNA	Noncoding RNA
lncRNA	Long noncoding RNA
TGF-β	Transforming growth factor beta
PDGF	Platelet-derived growth factor
VEGF	Vascular endothelial growth factor
FGF	Fibroblast growth factor
FGFR	Fibroblast Growth Factor Receptor
IGF	Insulin-like growth factor
cDNA	complementary DNA
ANGPT2	Angiopoietin2
PABPC1	Polyadenylate-binding protein cytoplasmic 1
GO	Gene ontology
KEGG	Kyoto Encyclopedia of Genes and Genomes
qRT-PCR	Quantitative real-time polymerase chain reaction
RPII	RNA polymerase II
LNAs	Lock nucleic acids
ASOs	Antisense oligonucleotides
